# Microwave-Assisted Extraction of Phenolic Compounds from Cocoa Pod Husk: Process Optimization and Impact of Drying Temperature on Bioactive Recovery

**DOI:** 10.3390/molecules30173497

**Published:** 2025-08-26

**Authors:** Pablo Gomez, Cristhopher Reyes, Jorge G. Figueroa

**Affiliations:** 1 Carrera de Ingeniería Química, Facultad de Ciencias Exactas y Naturales, Universidad Técnica Particular de Loja, París s/n y Praga, Loja 110107, Ecuador; psgomez1@utpl.edu.ec (P.G.); careyes19@utpl.edu.ec (C.R.); 2 Departamento de Química, Facultad de Ciencias Exactas y Naturales, Universidad Técnica Particular de Loja, París s/n y Praga, Loja 110107, Ecuador

**Keywords:** cocoa pod husk, microwave-assisted extraction, catechin, epicatechin, procyanidin B2, clovamide, response surface methodology, drying temperature, HPLC-DAD-ESI-IT-MS/MS

## Abstract

Cocoa pod husk (CPH), the principal by-product of cocoa processing, represents an abundant and underutilized source of bioactive phenolics with potential applications in the food and nutraceutical sectors. This study optimized the extraction of catechin, epicatechin, procyanidin B2, and clovamide from CPH (CCN-51 variety) using microwave-assisted extraction (MAE) and evaluated the influence of drying temperature on their retention. A Box–Behnken design within a response surface methodology framework was employed to evaluate the effects of ethanol concentration (0–100%), extraction temperature (50–150 °C), and extraction time (15–60 min) on compound recovery. The phenolic profile was characterized by high-performance liquid chromatography with diode-array detection and electrospray ionization ion trap tandem mass spectrometry. Optimal MAE conditions of 51% ethanol, 104 °C, and 38 min yielded maximum concentrations of clovamide, procyanidin B2, and epicatechin of 3440, 908, and 445 mg/kg dry matter of cocoa pod husk, respectively. Drying studies demonstrated that moderate hot-air temperatures (40–50 °C) preserved the highest phenolic levels. These results underscore the importance of optimizing both extraction and drying conditions to enhance the recovery of phenolic compounds from cocoa processing residues, supporting their potential valorization as antioxidant-rich functional ingredients.

## 1. Introduction

*Theobroma cacao* L., a member of the Malvaceae family, is a perennial tropical tree native to Central and South America [1]. The principal producers are Ivory Coast, Ghana, Indonesia, Ecuador, and Brazil [2]. Its seeds, known as cocoa beans, are valued for their distinctive flavor and nutritional properties, contributing significantly to the global economy [3]. In 2023, approximately 5.6 million metric tons of cocoa beans were processed worldwide [2]. This large-scale production generates considerable amounts of by-products, with approximately two kilograms produced for every kilogram of beans [4]. These by-products consist mainly of cocoa pod husk (CPH), mucilage, and bean shell, with CPH accounting for around 86% of the total biomass. Although traditionally underused, CPH contains high levels of phenolic bioactive compounds with documented antioxidant, anti-inflammatory, and antidiabetic properties [4,5,6]. These include protocatechuic acid, *p*-hydroxybenzoic acid, salicylic acid, kaempferol, linarin, resveratrol, apigenin, and luteolin [7,8], which confer significant potential for its valorization into functional ingredients. Nevertheless, due to its high moisture content (approximately 90%), CPH must first be dehydrated to prevent microbial spoilage and ensure material stability [3,5].

In this context, several drying techniques, including sun drying, freeze drying, vacuum drying, and forced convection drying, have been applied to CPH [4,6,9]. Sun drying, although inexpensive and widely accessible, is highly dependent on climatic conditions and presents an elevated risk of microbial contamination [4]. Freeze-drying ensures outstanding preservation of thermolabile compounds [10]. Nevertheless, its large-scale implementation is constrained by substantial capital requirements and high operational energy demand [5,8]. Vacuum drying reduces oxidative degradation but may result in heterogeneous moisture removal within the biomass [10]. Forced convection drying stands out among the available techniques because it allows precise control over temperature and airflow, which in turn ensures consistent moisture removal across the biomass [3,9]. Furthermore, forced convection drying is adaptable to different scales of operation, from laboratory to industrial levels, and offers shorter processing times while maintaining reproducibility and product quality [10]. These advantages make it a particularly attractive option for the stabilization of CPH, despite its significant energy requirements and dedicated infrastructure [10]. Nonetheless, most bioactive compounds are sensitive and prone to degradation when exposed to drying conditions such as high temperatures and oxygen-rich environments [11]. Therefore, it is crucial to evaluate the influence of drying temperature on their stability in order to establish conditions that safeguard both chemical integrity and functional properties [4].

Once stabilized through drying, efficient extraction becomes the next critical step, requiring methods that maximize yields while preserving compound integrity [12]. In this context, advanced techniques such as microwave-assisted extraction (MAE), pressurized liquid extraction, supercritical fluid extraction, and ultrasound-assisted extraction have been widely studied. These extraction methods are considered environmentally friendly, as they reduce solvent consumption and processing time. Nevertheless, each presents inherent technical and operational constraints. Pressurized liquid extraction and supercritical fluid extraction require high-pressure systems and high energy input, which restrict their industrial feasibility [13]. Ultrasound-assisted extraction may be effective for some matrices, but it often shows low efficiency for certain phenolic structures and can induce free radical formation that compromises stability [14]. MAE provides high efficiency and selectivity for phenolics, although it may cause non-uniform heating in heterogeneous matrices and increase the risk of thermal degradation of thermolabile compounds [15].

Among these alternatives, MAE stands out for its ability to use microwave energy to disrupt cellular matrices and promote the rapid release of intracellular compounds, typically with polar solvents that efficiently absorb microwave energy and enhance mass transfer [16]. MAE is also inherently adaptable to industrial-scale operations through appropriate reactor design and process optimization [17], making it a promising technology for large-scale phenolic recovery while preserving compound integrity. Given the potential and limitations of MAE, its successful application requires careful optimization of operational parameters to maximize phenolic recovery while preventing thermal degradation. In this context, Response Surface Methodology (RSM) has proven useful in cocoa matrices for defining suitable MAE conditions and improving extraction efficiency [18,19].

Although previous studies have demonstrated the potential of CPH as a rich source of phenolic compounds, most research has addressed extraction optimization or drying effects separately and has relied primarily on bulk phenolic measurements or qualitative identification of individual compounds without detailed molecular profiling. Rahayu, et al. [20], Van, et al. [21], and Mashuni, Hamid, Muzuni, Kadidae, Jahiding, Ahmad, and Saputra [5] evaluated MAE using ethanol–water mixtures, but their analyses were restricted to total phenolics and basic identification of catechin, without compound-specific quantification or multivariate statistical optimization. Other works have emphasized CPH valorization using alternative extraction methods such as sonotrode-assisted extraction or optimization of drying strategies such as vacuum or forced convection drying [6,8]. Nonetheless, none of these studies investigated how drying-induced transformations impact the recovery and structural stability of specific thermolabile compounds, including procyanidin B2 and clovamide, both of which are prone to enzymatic and thermal degradation. Furthermore, Cádiz-Gurrea, Fernández-Ochoa, Leyva-Jiménez, Guerrero-Muñoz, Villegas-Aguilar, Pimentel-Moral, Ramos-Escudero, and Segura-Carretero [3] provided only qualitative chemical identification without quantification or integration with process optimization frameworks. Therefore, this study aimed to optimize the extraction of phenolic compounds from CPH using MAE with generally recognized as safe (GRAS) solvents and to assess the effect of drying temperature on the recovery and stability of key bioactives, namely catechin, epicatechin, procyanidin B2, and clovamide.

## 2. Results and Discussion

### 2.1. Identification of Phenolic Compounds in Cocoa Pod Husk

Catechin and epicatechin, eluting at RT = 20.2 min and RT = 23.7 min, respectively, both exhibited [M−H]^−^ ions at *m*/*z* 289.2 and identical characteristic fragments at *m*/*z* 165, 179, 245, and 271 (Table 1). These fragment ions result from typical retro-Diels–Alder cleavage and heterocyclic ring fission processes associated with the C-ring of flavan-3-ols, consistent with previously reported fragmentation profiles for catechin and epicatechin [9].

Procyanidin B2, detected at RT = 20.9 min, was identified by its [M−H]^−^ ion at *m*/*z* 577.3 and diagnostic fragments at *m*/*z* 205, 289, 425, and 559 (Table 1). These signals correspond to sequential quinone methide and heterocyclic ring cleavages, including the loss of catechin/epicatechin monomer units, confirming its structure as a B-type procyanidin dimer [22].

Clovamide, eluting at RT = 28.4 min (Table 1), was tentatively identified as [M−H]^−^ at *m*/*z* 358.2. Its MS/MS fragments at *m*/*z* 178, 222, and 314 reflect characteristic cleavages of its caffeic acid and L-DOPA-derived substructures, as previously described for phenolic amides in cocoa-related matrices [8].

### 2.2. Optimization of Phenolic Compound Extraction by MAE

Extraction is a critical step in maximizing the recovery of target compounds from plant matrices [8]. Several factors influence the efficiency of the extraction process, including the extraction method, solvent type, temperature, extraction time, and solvent-to-sample ratio, as well as sample-related characteristics such as fat content, moisture level, and particle size [23]. In this study, ethanol concentration, extraction temperature, and extraction time were selected as independent variables, while solvent volume, sample mass, and particle size were kept constant.

MAE was chosen due to its advantages over conventional extraction methods. MAE offers shorter extraction times, reduced solvent consumption, higher extraction efficiency, and lower processing costs [15].

With regard to the solvent system, ethanol and water were selected as extraction media, both of which are classified as GRAS and therefore suitable for use in the food and nutraceutical industries [13]. Moreover, the combination of ethanol and water has been shown to enhance the extraction efficiency of phenolic acids and flavonoids [13].

Temperature is another key factor, as elevated temperatures promote the rupture of plant cell structures, facilitating the release of phenolic compounds and improving extraction yields [24]. Nevertheless, excessively high temperatures should be avoided due to the thermal sensitivity of polyphenols, which are prone to degradation under such conditions [12]. Accordingly, extraction temperature was evaluated in the range of 50 to 150 °C.

Extraction time also plays a critical role in MAE, a technique known for its ability to heat the sample uniformly both internally and externally without thermal gradients. As a result, extraction can be achieved in shorter durations [12]. For this reason, extraction time was evaluated within the range of 15 to 60 min.

The response variables selected for optimization were the concentrations of procyanidin B2, catechin, epicatechin, and clovamide. Notably, procyanidins, catechin, and epicatechin are flavonoids belonging to the polyphenol family. These compounds possess antioxidant capacities 20 to 50 times higher than those of vitamins C and E, respectively [25]. In addition to their antioxidant potential, they have demonstrated various health-promoting effects, including glycemic and insulin regulation, neuroprotective properties, anti-tumor activity, and anti-inflammatory effects [26].

Regarding clovamide, this compound is of particular interest due to its notable antioxidant activity. It has been shown to exhibit free radical scavenging properties comparable to those of rosmarinic acid and caffeic acid and to be more effective than other well-known antioxidants such as epicatechin [27]. These findings highlight clovamide as a promising bioactive compound for pharmaceutical and food-related research, with potential applications as a nutraceutical ingredient.

The BBD, integrated within the framework of RSM, was employed to optimize the extraction of phenolic compounds from cocoa by-products and to evaluate the combined effects of ethanol concentration, extraction temperature, and extraction time. This approach enables efficient analysis of multiple variables while minimizing the number of experimental runs, time, and material consumption. To assess the adequacy of the mathematical model, an analysis of variance (ANOVA) was performed, followed by Fisher’s statistical test to determine the significance of each independent variable.

The adequacy and reliability of the statistical models obtained for the extraction process were evaluated through ANOVA. The resulting quadratic models exhibited R^2^ ranging from 73% to 91%, indicating excellent agreement between experimental data and predicted values and confirming the suitability of these models for predicting extraction behavior [28].

Ethanol concentration showed a pronounced quadratic effect (*p* < 0.05) on the extraction yield of all four compounds, clearly demonstrating optimal points within the tested range (0–100%). This observation highlights the critical role that ethanol-water mixtures play in phenolic compound extraction. Specifically, intermediate ethanol concentrations (~50%) consistently produced the highest yields, which can be attributed to the synergistic solvation properties of binary solvent systems. Such mixtures improve the polarity balance of the extraction medium, facilitating the solubilization of both hydrophilic and moderately lipophilic phenolics, enhancing mass transfer, and helping to stabilize thermolabile compounds during extraction [29].

**Figure 1:**
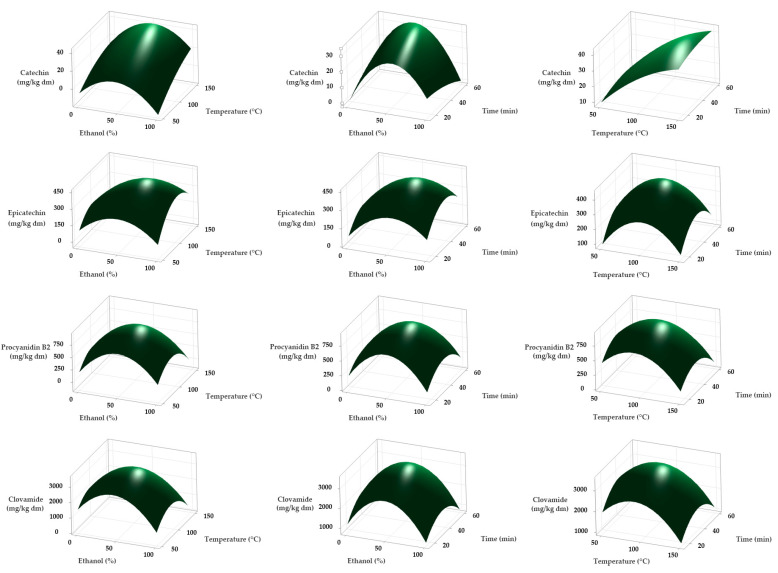
Response surface plots showing the effects of ethanol concentration (%), extraction temperature (°C), and extraction time (min) on the concentration of phenolic compounds from cocoa pod husk: catechin, epicatechin, procyanidin B2, and clovamide.

The response surface plots (Figure 1) and regression models demonstrated a significant negative quadratic effect (*p* < 0.05) of ethanol concentration on the recovery of catechin, epicatechin, procyanidin B2, and clovamide, with coefficients of −0.00949, −0.0668, −0.1979, and −0.654, respectively. These results indicate that extraction efficiency peaked at intermediate ethanol–water ratios and declined toward both extremes of the tested range, a behavior consistent with the influence of solvent polarity on phenolic extraction.

Hydroethanolic mixtures at intermediate proportions are more effective than single solvents because their balanced polarity improves the solubility of compounds with different polarities, while the aqueous fraction facilitates desorption of analytes from the plant matrix. This combination of solvent effects has been explicitly reported for MAE and related techniques [12,26].

This interpretation is consistent with findings from other MAE studies in various plant matrices, where ethanol–water mixtures around 45–60% yielded higher total phenolic contents than either pure water or pure ethanol [19,29]. The agreement with published data from other polyphenol-rich sources supports the conclusion that intermediate ethanol concentrations provide an optimal polarity balance, improving extraction yield while preserving compound integrity.

Extraction temperature significantly influenced the recovery of the target polyphenols, although the nature of the effect differed among compounds. For epicatechin, procyanidin B2, and clovamide, regression models revealed a significant quadratic term for temperature (*p* = 0.003, *p* = 0.005, and *p* = 0.019, respectively), indicating that maximum extraction efficiency occurred at intermediate values. Within this range, MAE promotes efficient solvent penetration and cell disruption while limiting degradation, as the rapid dielectric heating of the intracellular water causes cell wall rupture and accelerates the release of phenolic compounds without prolonged exposure to thermal stress [30]. Beyond this optimum, concentrations declined markedly, consistent with the negative quadratic coefficients and the known thermolability of these compounds.

Procyanidin B2, a dimeric flavan-3-ol composed of two epicatechin units linked by an interflavan C4–C8 bond, is particularly susceptible to thermal depolymerization [25]. Moderate heating may liberate monomeric units, but further heating leads to oxidative degradation, a pathway more pronounced than in the monomeric catechin. Catechin showed no significant quadratic temperature effect (*p* = 0.572) but exhibited a positive linear relationship (*p* = 0.039), suggesting that the apparent steady increase in catechin concentration within the tested range may be partially explained by the thermal depolymerization of procyanidin B2, which releases monomeric epicatechin units that can undergo epimerization to catechin before significant degradation occurs. Epicatechin, despite also being a monomer, displayed a pronounced quadratic response due to its higher susceptibility to epimerization, oxidative cleavage, and condensation reactions under heat stress [11]. Catechin and epicatechin, although structural isomers, showed distinct thermal behaviors under MAE in this study. Catechin exhibited a positive trend, consistent with formation from procyanidin depolymerization and epimerization of epicatechin, whereas epicatechin peaked at intermediate temperatures before declining due to greater reactivity. Liazid, et al. [31] reported a similar divergence: catechin remained stable up to 100 °C and increased further at 125 °C and 150 °C, likely due to liberation from oligomers, while epicatechin stayed stable up to 100 °C but did not show a comparable increase at higher temperatures, reflecting differences in reactivity despite structural similarity.

Thermal degradation of epicatechin, procyanidin B2, and clovamide involves oxidative cleavage, depolymerization, and structural rearrangements that reduce the concentration of individual compounds in the extract. Comparable effects have been reported in polyphenol-rich matrices such as grape seed and cocoa bean extracts, where excessive heating markedly reduced total procyanidin content [19,31,32]. These findings highlight the importance of optimizing MAE temperature conditions according to the thermal stability and structural characteristics of each phenolic compound to maximize yield while preserving their integrity.

The response surface plots (Figure 1) and regression analyses showed that the influence of extraction time under MAE conditions varied markedly among the phenolic compounds analyzed. For catechin and clovamide, neither the linear nor the quadratic terms for time were statistically significant (*p* > 0.07), indicating that extraction equilibrium was reached rapidly. This suggests that prolonged extraction times offer no measurable advantage for their recovery. In the case of clovamide, the quadratic model was significant overall (*p* = 0.044) due to pronounced curvature effects of solvent composition and temperature, whereas time contributed minimally to model performance, with the quadratic effect of time showing only marginal significance (*p* = 0.071). This trend aligns with the fundamental principles governing MAE, where the ability to heat the sample matrix both internally and externally without a thermal gradient accelerates cell disruption and solute release, thereby achieving maximum yields in the first minutes of extraction [15,16].

In contrast, epicatechin and procyanidin B2 exhibited statistically significant quadratic effects of time (*p* = 0.008 and *p* = 0.024, respectively), resulting in a parabolic response within the tested range (20–60 min). Maximum yields were obtained at intermediate extraction times, while both shorter and longer durations produced lower recoveries. This pattern reflects a balance between rapid solubilization facilitated by dielectric heating and subsequent degradation, epimerization, or reduced mass transfer efficiency when extraction is excessively prolonged [12]. Comparable trends have been reported for MAE of phenolics from other plant matrices. For instance, in *Agaricus blazei* Murrill, extraction yields of several phenolic compounds reached a maximum at intermediate times and subsequently declined, a behavior attributed to thermal degradation and structural transformations during prolonged microwave exposure [29].

The differences in time dependence are likely related to molecular structure and stability. Procyanidin B2 contains multiple hydroxyl groups that are more susceptible to oxidative or thermal degradation [25]. Being a dimeric flavanol, it may also undergo depolymerization under extended microwave exposure. In contrast, clovamide possesses an amide linkage and conjugated aromatic system that confer greater resistance to microwave-induced dielectric and thermal stress.

Overall, the statistical models confirmed that extraction time was a secondary factor compared with solvent composition and temperature for all analytes except epicatechin and procyanidin B2. For catechin and clovamide, short extraction times are sufficient, while intermediate durations are optimal for epicatechin and procyanidin B2 to balance rapid release with minimal degradation. These results support the widely recognized advantage of MAE in achieving high phenolic recoveries in significantly shorter times than conventional methods, driven by its dielectric heating mechanism and uniform energy distribution within the extraction matrix [19,31].

**Figure 2:**
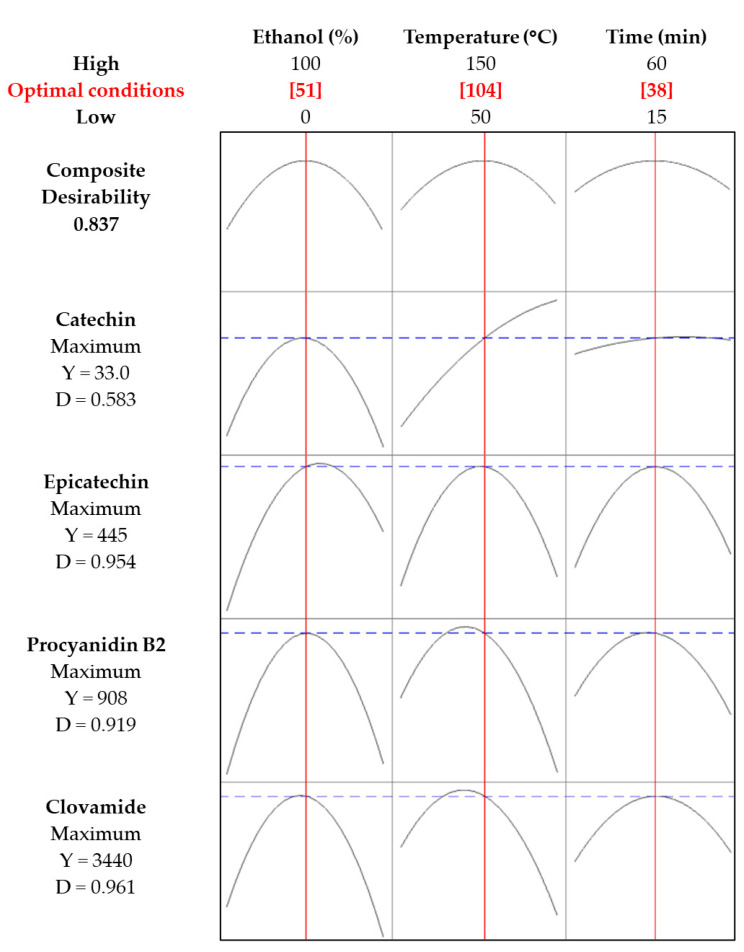
Response optimizer plot showing the desirability-based optimization of ethanol concentration (%), extraction temperature (°C), and extraction time (min) for the microwave-assisted extraction of catechin, epicatechin, procyanidin B2, and clovamide from cocoa pod husk. The red vertical lines indicate the optimal conditions (51% ethanol, 104 °C, 38 min) for maximizing the individual responses and the overall composite desirability (0.837). For each compound, Y represents the predicted maximum concentration achieved at the optimal conditions, while D denotes the individual desirability value (ranging from 0 to 1), indicating how closely the predicted concentration meets the optimization target, with 1 representing the ideal value.

The composite desirability and individual desirability profiles (Figure 2) identified the optimal MAE conditions as 51% ethanol, 104 °C, and 38 min, yielding a composite desirability of 0.837. Clovamide and procyanidin B2 achieved the highest desirabilities (0.961 and 0.919, respectively), corresponding to yields of 3440 mg/kg dm and 908 mg/kg dm. Epicatechin exhibited a desirability of 0.954 with a yield of 445 mg/kg dm, aligning with findings by Maldonado and Figueroa [19], who showed that epicatechins remain stable and well-recovered under moderate-temperature MAE with ethanol-water mixtures and degrade only at excessive heat. In contrast, catechin showed the lowest desirability (0.583) and yield of 33.0 mg/kg dm.

This outcome likely reflects its lower initial concentration in CPH and its sensitivity to extraction temperature. Notably, catechin levels increased at higher temperatures, probably as a result of the thermal depolymerization of higher procyanidin oligomers (dimers, trimers, and tetramers) into monomeric catechin units.

This finding indicates that although elevated temperatures may indirectly promote catechin recovery, they introduce challenges in defining the overall optimal extraction conditions.

The optimized MAE parameters were subsequently employed to assess the effect of drying temperature on the phenolic composition of CPH.

### 2.3. Effect of Drying Temperature on Phenolic Content

**Figure 3:**
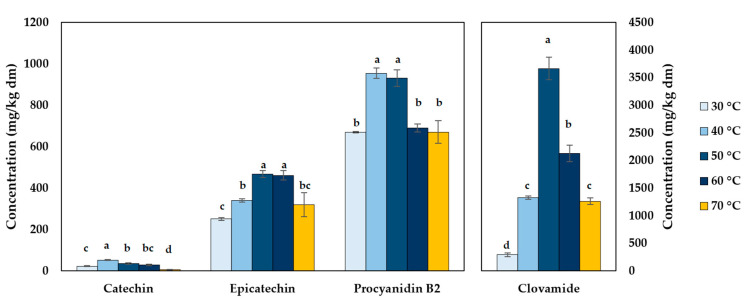
Effect of drying temperature on the concentration of phenolic compounds (catechin, epicatechin, procyanidin B2, and clovamide) in cocoa pod husk. Different letters above bars indicate significant differences among temperatures for each compound (Tukey’s test, *p* < 0.05).

Drying temperature exerted a pronounced influence on the retention of catechin, epicatechin, procyanidin B2, and clovamide in CPH (Figure 3). Maximum phenolic concentrations were consistently observed under moderate hot-air drying (40–50 °C), with statistically significant differences among temperatures for all analytes (Tukey’s test, *p* < 0.05). 

The combined concentration of the four quantified phenolics reached a maximum of 5099 mg/kg dm at 50 °C. In contrast, the lowest totals were observed at 30 °C (1235 mg/kg dm) and 70 °C (2255 mg/kg dm), representing 75.8% and 55.8% reductions, respectively, compared with the maximum. Moderate drying temperatures inactivate degradative enzymes such as polyphenol oxidase and peroxidase [33]. This temperature range also improves extractability by disrupting cell wall integrity and weakening the polysaccharide–protein network, which increases solvent access and facilitates phenolic release [34]. At ≥ 60–70 °C, non-enzymatic oxidative and thermal reactions increase, causing the loss of flavan-3-ols and hydroxycinnamic acid amides [35]. At 30 °C, prolonged moisture retention allows enzymatic oxidation to proceed [1]. These trends agree with reports on CPH and other cocoa tissues dried with hot air or microwaves, where intermediate temperatures favor phenolic retention [9].

Clovamide was the most abundant phenolic, peaking at 3665 ± 184 mg/kg dm at 50 °C. Its concentration increased markedly from 293 ± 34 mg/kg dm at 30 °C to 1325 ± 31 mg/kg dm at 40 °C, before reaching its maximum at 50 °C. Beyond this point, levels dropped significantly to 2126 ± 140 mg/kg dm at 60 °C and 1261 ± 61 mg/kg dm at 70 °C. This biphasic behavior reflects clovamide’s dual susceptibility to degradation. At lower temperatures, polyphenol oxidase-mediated oxidation of its caffeic acid moiety likely drives quinone formation and polymerization [36]. At higher temperatures, non-enzymatic decarboxylation and free-radical oxidation predominate, fragmenting the caffeic acid structure and diminishing bioactivity [27]. These findings indicate that drying at ~50 °C is optimal to preserve clovamide, whose antioxidant potency makes it valuable for nutraceutical applications.

Procyanidin B2 peaked at 952 ± 44 mg/kg dm at 40–50 °C, with concentrations significantly lower at 30 °C (670 ± 2 mg/kg dm) and dropping again at 70 °C (670 ± 52 mg/kg dm). The moderate accumulation at 40–50 °C suggests enhanced extractability without critical structural degradation, while losses at 70 °C are attributable to cleavage of the interflavan bond and oxidative conversion into procyanidin A2 and catechin units, as documented in model systems heated at 60–90 °C [37]. These oligomeric flavan-3-ols, although relatively stable compared to clovamide, are prone to oxidative depolymerization under heat, which not only liberates monomeric catechin and epicatechin but also generates insoluble, polymerized residues, reducing the pool of recoverable bioactives.

Epicatechin showed maximal retention at 50 °C (454 ± 21 mg/kg dm), maintaining high levels at 40 °C (340 ± 6 mg/kg dm) and 60 °C (461 ± 22 mg/kg dm) but declining sharply to 319 ± 47 mg/kg dm at 70 °C. Its cis-stereochemistry renders epicatechin particularly prone to epimerization into catechin and to oxidative condensation at elevated temperatures [38]. This epimerization process, accelerated by microwaves or thermal stress, has been reported to yield dimers and trimers, particularly at 60–90 °C in aqueous systems [37]. Such transformations reduce free epicatechin but can transiently enrich monomeric catechin pools.

Catechin exhibited relatively low concentrations across all drying treatments, reaching a maximum of 51 ± 2 mg/kg dm at 40 °C before declining sharply to near-undetectable levels (4 ± 2 mg/kg dm) at 70 °C. Moderate thermal exposure favors catechin formation through the depolymerization of higher-order procyanidins, such as dimers and trimers, under microwave or hot-air conditions [32]. Nevertheless, at temperatures of 70 °C and above, this generation is offset by accelerated degradation through multiple pathways. Catechin can epimerize back to epicatechin or condense into oligomeric species, including dimers, trimers, and dehydrodi(epi)catechin A [37]. Additionally, its monomeric form is highly prone to oxidative polymerization under oxygen-rich hot-air drying, resulting in the formation of insoluble, high-molecular-weight complexes that are not quantified as free catechin [39]. These degradation pathways collectively surpass catechin formation from procyanidin cleavage at high temperatures, explaining the pronounced reduction in its measurable concentration at 70 °C.

Collectively, these results indicate that 40–50 °C represents the optimal drying range for CPH, balancing the extractability of key phenolics with minimal degradation. Higher temperatures (≥60 °C) accelerate non-enzymatic oxidation and interflavan cleavage, while lower temperatures (≤30 °C) favor enzymatic browning, both of which diminish the availability of bioactive compounds.

## 3. Materials and Methods

### 3.1. Chemicals and Reagents

Ethanol (analytical grade) for extraction was purchased from Kimia (Witham, UK). LC-MS grade water was obtained from Merck KGaA (Darmstadt, Germany). HPLC-grade acetonitrile was sourced from Fisher Chemical (Mumbai, India). Analytical standards, including catechin, epicatechin, procyanidin B2, and caffeic acid, were supplied by Sigma-Aldrich (St. Louis, MO, USA) and LGC Standards (Toronto, ON, Canada).

### 3.2. Raw Material

Samples of the CCN-51 cocoa variety were purchased in March 2022 from a local producer in the canton of Ventanas, Los Ríos Province, Ecuador. The pods were thoroughly washed with water to remove surface impurities and allowed to rest for four days to facilitate the natural detachment of the mucilage-coated beans from the pod husk. Each pod was first split longitudinally through the center to remove the beans, and each half was then cut into approximately four sections, resulting in a total of about eight segments per pod. The husk pieces were immediately subjected to the drying process.

### 3.3. Dehydration Process

CPH was dried at 30 °C, 40 °C, 50 °C, 60 °C, and 70 °C using a hot-air dryer (DY-330H, Lassele, Ansan, Republic of Korea). The samples were periodically turned to ensure uniform exposure to airflow, and drying was continued until the moisture content was reduced to below 10%. The drying times required to reach this target were approximately 62, 50, 39, 30, and 23 h, respectively. The final moisture content of each sample was determined using a moisture analyzer (HE73-03, Mettler Toledo, Columbus, OH, USA). The dried husks were subsequently ground using an ultra-centrifugal mill (ZM 200, Retsch GmbH, Haan, Germany) and sieved with a vibratory sieve shaker (AS 200, Retsch GmbH, Haan, Germany) to obtain particles within a size range of 250–500 µm. All drying experiments were conducted in triplicate. The resulting CPH powder was stored at room temperature, protected from light, until further extraction and analysis.

### 3.4. Experimental Design

MAE was optimized using RSM. A BBD with three factors and three levels (3^3^) was employed, where extraction temperature (50–150 °C), ethanol concentration (0–100%), and extraction time (15–60 min) were selected as the independent variables. For all experimental runs, CPH previously dried at 50 °C was used, and the solid-to-solvent ratio (1:10) and particle size (250–500 µm) were kept constant for all experiments. The concentrations of catechin, epicatechin, procyanidin B2, and clovamide were used as response variables. The experimental design comprised 15 randomized runs, including replicated central points to estimate pure error and evaluate lack of fit, as detailed in Table 2. Randomization was applied to minimize uncontrolled variability. Analysis of variance (ANOVA) followed by Fisher’s test was used to determine the statistical significance of main effects and interactions. The adequacy of the fitted models was assessed based on the coefficient of determination (R^2^) and lack-of-fit tests.

### 3.5. Microwave-Assisted Extraction

For each extraction, 2 g of previously dried CPH was placed in 20 mL of solvent inside 40 mL Teflon vessels. The 50 °C drying temperature was selected as it effectively reduces moisture content while limiting thermal degradation of phenolic compounds, consistent with previous studies on cocoa by-products [6]. Extractions were carried out in a laboratory microwave system (Mars 6, CEM Corporation, Matthews, NC, USA) operating at 1200 W, with a 10 min ramp period to reach the target extraction temperature. Once the extraction time had elapsed, the system was programmed for a 15 min cooling phase before the vessels were removed. Immediately afterward, the vessels were immersed in an ice-water bath to prevent degradation of thermolabile compounds. The extracts were then transferred to 50 mL centrifuge tubes and centrifuged at 8500 rpm for 20 min using a centrifuge (Sorvall ST8, Thermo Fisher Scientific, Waltham, MA, USA). The resulting supernatants were collected and stored at −20 °C until further analysis.

### 3.6. Quantification of Phenolic Compounds by HPLC-DAD-ESI-IT-MS/MS

All extracts obtained from the microwave-assisted extraction experiments, as well as samples from each drying temperature evaluated under the optimal extraction conditions, were analyzed by HPLC–DAD–ESI–IT–MS/MS. Prior to chromatographic analysis, supernatants were filtered through 0.2 µm Choice™ regenerated cellulose syringe filters (CH2225-RC, Thermo Scientific, Waltham, MA, USA) to remove particulate matter. Bioactive compounds from the cocoa by-product were analyzed using a high-performance liquid chromatography system (Dionex Ultimate 3000, Thermo Scientific, Waltham, MA, USA) coupled to an ion trap mass spectrometer with an electrospray ionization (ESI) source (AmaZon Speed, Bruker Daltonics, Billerica, MA, USA). Chromatographic separation was performed on a C18 reversed-phase column (Acclaim™ 120, 250 mm × 4.6 mm, 5 µm; Thermo Fisher Scientific, Waltham, MA, USA) maintained at 25 °C, following the procedure described by Maldonado and Figueroa [19]. The mobile phase consisted of acetonitrile (MeCN) and water (H_2_O), using a decreasing polarity gradient at a constant flow rate of 0.5 mL/min: 0–3 min, 5% MeCN; 3–10 min, linear increase to 18% MeCN; held at 18% until 19 min; 19–20 min, increased to 21% MeCN; 20–50 min, linear increase to 30% MeCN; 50–60 min, linear increase to 60% MeCN; 60–61 min, held at 60% MeCN; and 61–66 min, decreased to 5% MeCN. The injection volume was 10 µL, and detection was performed with a photodiode array detector (Thermo Scientific, Waltham, MA, USA) at 280 nm.

The system was controlled using Bruker Daltonics (Bremen, Germany) HyStar 3.2 software with an Ethernet data interface. Mass spectrometry was performed in negative ionization mode under the following conditions: capillary voltage 4.500 V; *m*/*z* range 100–2000; nebulizer pressure 26.0 psi; nitrogen drying gas temperature 200 °C; drying gas flow 6.0 L/min; rolling averages set to 2 counts; number of averages set to 2; and ion charge control activated.

Catechin, epicatechin, and procyanidin B2 were quantified using external standards, while clovamide was expressed as caffeic acid equivalents due to their structural and ionization similarities, in accordance with established phytochemical practices [40]. Calibration curves for all compounds were constructed with six concentration levels and yielded R^2^ ≥ 0.995. The limit of quantification, determined from standard solutions, was 1.0 mg/kg and corresponded to the lowest concentration that yielded an acceptable coefficient of determination (R^2^) in the calibration curve.

### 3.7. Statistical Analysis

Once the optimal extraction conditions were established, the effect of drying temperature on polyphenol content was evaluated. For this purpose, ANOVA was performed, followed by Tukey’s multiple range test for post hoc comparisons. All statistical analyses were conducted using Minitab software, version 14.0 (Minitab Inc., State College, PA, USA).

## 4. Conclusions

MAE of phenolic compounds from CPH was successfully optimized using response surface methodology, identifying 51% ethanol, 104 °C, and 38 min as the optimal conditions. Under these parameters, clovamide, procyanidin B2, and epicatechin were efficiently recovered, whereas catechin levels were largely influenced by the thermal depolymerization of procyanidins. Drying temperature exerted a significant effect on phenolic retention, with 40–50 °C identified as the optimal range to simultaneously achieve enzymatic inactivation and minimize thermal degradation. These findings establish a robust process framework for the valorization of CPH as a sustainable source of antioxidant-rich phenolics with potential applications in functional foods and nutraceuticals.

Future research should focus on enhancing extract stability and expanding its applications in food systems. Microencapsulation strategies, including spray-drying, freeze-drying, and complex coacervation, should be further explored to protect phenolic compounds during processing and storage. In parallel, alternative approaches such as nanoemulsions, edible coatings, and direct incorporation in powdered form could provide versatile options for functional ingredient development. The extract may also be incorporated into beverages, bakery products, dairy, and confectionery, where phenolics can act as natural antioxidants or bioactive enhancers. Evaluating these applications under realistic processing and storage conditions will offer valuable insight into technological feasibility and functional performance. Additionally, bioaccessibility and bioavailability studies, employing simulated gastrointestinal digestion and Caco-2 cell assays, will be essential to confirm physiological relevance. Another promising avenue is the isolation of clovamide, procyanidin B2, catechin, and epicatechin using flash chromatography or preparative HPLC. This would enable targeted investigations into their bioactivity, stability, synergistic interactions, and bioavailability. Finally, integrating MAE into a sequential biorefinery for the co-recovery of pectin could enhance both economic viability and sustainability in CPH valorization.

## Figures and Tables

**Table 1:** Mass spectrometric characteristics of phenolic compounds identified in cocoa pod husk by high-performance liquid chromatography with diode-array detection and electrospray ionization ion-trap tandem mass spectrometry.

Compound	RT (min)	*m*/*z*	MS/MS Fragments
Catechin	20.2	289.2	165, 179, 245, 271
Procyanidin B2	20.9	577.3	205, 289, 425, 559
Epicatechin	23.7	289.2	165, 179, 245, 271
Clovamide	28.4	358.2	178, 222, 314

**Table 2:** Experimental matrix of the Box–Behnken Design showing the effect of ethanol concentration, extraction temperature, and extraction time on the concentration of phenolic compounds (catechin, epicatechin, procyanidin B2, and clovamide) in cocoa pod husk expressed as mg/kg dry matter obtained by microwave-assisted extraction.

Run	Ethanol (%)	Temperature (°C)	Time (min)	Clovamide (mg/kg dm)	Procyanidin B2 (mg/kg dm)	Epicatechin (mg/kg dm)	Catechin (mg/kg dm)
1	50	100	37.5	3471	929	413	31
2	100	100	60	613	130	199	<LOQ
3	0	100	60	1328	25	101	26
4	100	100	15	888	200	107	<LOQ
5	50	100	37.5	3397	845	466	40
6	0	50	37.5	816	36	12	<LOQ
7	0	100	15	1361	283	96	<LOQ
8	50	100	37.5	3570	988	454	27
9	100	50	37.5	305	54	54	<LOQ
10	0	150	37.5	808	<LOQ	<LOQ	<LOQ
11	50	50	15	2441	518	153	<LOQ
12	100	150	37.5	845	80	308	15
13	50	150	15	238	<LOQ	74	57
14	50	150	60	783	<LOQ	46	44
15	50	50	60	2672	565	160	<LOQ

<LOQ: concentrations below the limit of quantification.

## Data Availability

All data used in this study are available upon request from the corresponding author.

## Abbreviations

The following abbreviations are used in this manuscript:

ANOVA	Analysis of variance
BBD	Box–Behnken Design
CPH	Cocoa pod husk
DAD	Diode-Array Detector
dm	Dry matter
ESI	Electrospray Ionization
HPLC	High-Performance Liquid Chromatography
IT	Ion Trap
LOQ	Limit of quantification
MAE	Microwave-assisted extraction
MS/MS	Tandem mass spectrometry
*m*/*z*	Mass-to-charge ratio
R^2^	Coefficients of determination
RSM	Response Surface Methodology
RT	Retention time
